# (2-Amino­phen­yl)(*p*-tol­yl)methanone

**DOI:** 10.1107/S1600536810049147

**Published:** 2010-11-30

**Authors:** Dun-Lin Zhang, Shan Liu, Xiao-Li Zhang

**Affiliations:** a School of Bio-chemistry and Environmental Engineering, Nanjing Xiaozhuang University, 3601 Hongjing Road, Jiangning District, Nanjing 211171, People’s Republic of China; b Department of Chemical Engineering, Nanjing College of Chemical Technology, No 625, Geguan Road, Luhe, Nanjing 210048, People’s Republic of China

## Abstract

In the title compound, C_14_H_13_NO, the two six-membered rings make a dihedral angle of 52.8 (3)°. An intra­molecular N—H⋯O hydrogen bond involving an amine H atom and the adjacent carbonyl O atom occurs. In the crystal, N—H⋯O and C—H⋯N inter­molecular hydrogen bonds are observed, which may be effective in stabilizing the structure.

## Related literature

For the uses of 5-nitro­thio­phene-2-carb­oxy­lic acid, see: Shetty *et al.* (1999 ▶). For the synthesis of the title compound, see: Zhu *et al.* (2005 ▶). For standard bond-length data, see: Allen *et al.* (1987 ▶).
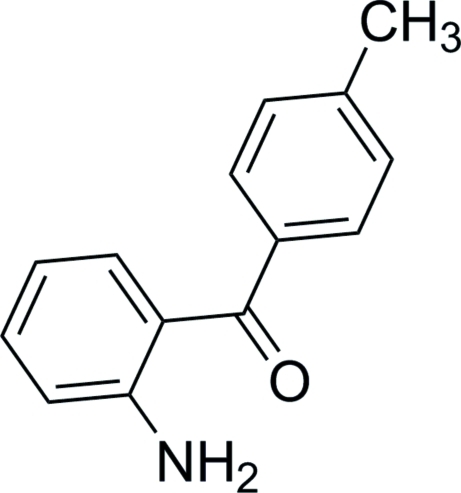


## Experimental

### 

#### Crystal data

C_14_H_13_NO
*M* *_r_* = 211.25
Orthorhombic, 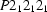

*a* = 7.7720 (16) Å
*b* = 10.490 (2) Å
*c* = 14.114 (3) Å
*V* = 1150.7 (4) Å^3^
*Z* = 4
Mo *K*α radiation
μ = 0.08 mm^−1^
*T* = 298 K
0.30 × 0.20 × 0.10 mm

#### Data collection

Enraf–Nonius CAD-4 diffractometer
Absorption correction: ψ scan (North *et al.*, 1968 ▶) *T*_min_ = 0.977, *T*_max_ = 0.992
2387 measured reflections
1241 independent reflections
984 reflections with *I* > 2σ(*I*)
*R*_int_ = 0.023
3 standard reflections every 200 reflections  intensity decay: 1%

#### Refinement

*R*[*F* ^2^ > 2σ(*F* ^2^)] = 0.040
*wR*(*F* ^2^) = 0.114
*S* = 1.01
1241 reflections
154 parameters
H atoms treated by a mixture of independent and constrained refinement
Δρ_max_ = 0.15 e Å^−3^
Δρ_min_ = −0.12 e Å^−3^

### 

Data collection: *CAD-4 Software* (Enraf–Nonius, 1989 ▶); cell refinement: *CAD-4 Software*; data reduction: *XCAD4* (Harms & Wocadlo, 1995 ▶); program(s) used to solve structure: *SHELXS97* (Sheldrick, 2008 ▶); program(s) used to refine structure: *SHELXL97* (Sheldrick, 2008 ▶); molecular graphics: *SHELXTL* (Sheldrick, 2008 ▶); software used to prepare material for publication: *SHELXTL*.

## Supplementary Material

Crystal structure: contains datablocks I, global. DOI: 10.1107/S1600536810049147/su2231sup1.cif

Structure factors: contains datablocks I. DOI: 10.1107/S1600536810049147/su2231Isup2.hkl

Additional supplementary materials:  crystallographic information; 3D view; checkCIF report

## Figures and Tables

**Table 1:** Hydrogen-bond geometry (Å, °)

*D*—H⋯*A*	*D*—H	H⋯*A*	*D*⋯*A*	*D*—H⋯*A*
N1—H0*A*⋯O1	0.87 (3)	2.08 (3)	2.723 (4)	131 (3)
N1—H0*B*⋯O1^i^	0.82 (3)	2.45 (3)	3.220 (4)	158 (3)
C11—H11*A*⋯O1^i^	0.93	2.53	3.319 (4)	143

Symmetry code: (i) 
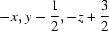
.

**Fig. 1.**
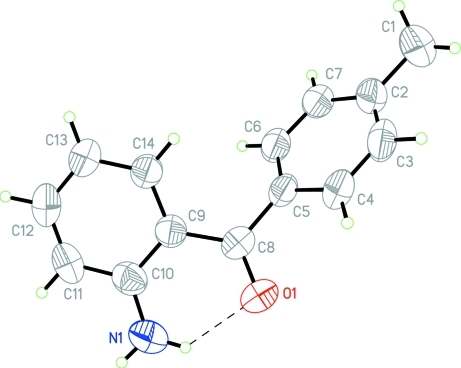
The molecular structure of the title compound, with the atom-numbering scheme. Displacement ellipsoids are drawn at the 50% probability level [The intermolecular N-H···O hydrogen bond is shown as a dashed line - Table 1].

**Fig. 2.**
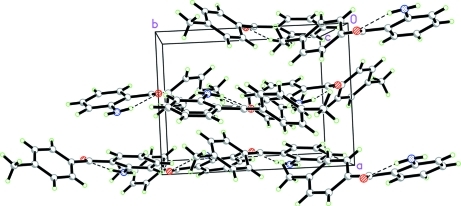
Crystal packing of the title compound viewed along the c-axis [The hydrogen bonds are shown as dashed lines; details are given in Table 1].

## supplementary crystallographic information

### Comment

(2-Aminophenyl)(*p*-tolyl)methanone and its derivitives are important monomers, being utilized to synthesize oligomers containing a quinoline unit (Shetty *et al.*, 1999). We report herein on the crystal structure of the title compound, (2-Aminophenyl)(*p*-tolyl)methanone.

In the title molecule (Fig. 1) the bond lengths (Allen *et al.*, 1987) and angles are within normal ranges. An amine H-atom and the adjacent carbonyl O-atom forms an intramolecular N-H···O hydrogen bond (Fig. 1, Table 1). The two aromatic rings are planar, with a dihedral angle of 52.8 (3)°.

In the crystal, N-H···O and C—H···N intermolecular hydrogen bonds are observed, which stabilize the crystal structure (Fig. 2, Table 1).

### Experimental

(2-Aminophenyl)(*p*-tolyl)methanone was prepared by the method reported in the literature (Zhu *et al.*, 2005). Single crystals were obtained by dissolving (2-aminophenyl)(*p*-tolyl)methanone (0.5 g, 2.37 mmol) in ethyl acetate (50 ml) and evaporating the solvent slowly at room temperature for about 10 d.

### Refinement

In the final cycles of refinement, in the absence of significant anomalous scattering effects, Friedel pairs were merged and Δf " set to zero. After checking their presence in a difference map, the NH_2_ H-atoms were freely refined. The C-bound H-atoms were positioned geometrically [C—H = 0.93 Å] and constrained to ride on their parent atoms, with *U*_iso_(H) = 1.2U_eq_(C).

### Crystal data

**Table:**

C_14_H_13_NO	*F*(000) = 448
*M**_r_* = 211.25	*D*_x_ = 1.219 Mg m^−^^3^
Orthorhombic, *P*2_1_2_1_2_1_	Mo *K*α radiation, λ = 0.71073 Å
Hall symbol: P 2ac 2ab	Cell parameters from 25 reflections
*a* = 7.7720 (16) Å	θ = 9–14°
*b* = 10.490 (2) Å	µ = 0.08 mm^−^^1^
*c* = 14.114 (3) Å	*T* = 298 K
*V* = 1150.7 (4) Å^3^	Plate, brown
*Z* = 4	0.30 × 0.20 × 0.10 mm

### Data collection

**Table:**

Enraf–Nonius CAD-4 diffractometer	984 reflections with *I* > 2σ(*I*)
Radiation source: fine-focus sealed tube	*R*_int_ = 0.023
graphite	θ_max_ = 25.4°, θ_min_ = 2.4°
ω/2θ scans	*h* = 0→9
Absorption correction: ψ scan (North *et al.*, 1968)	*k* = 0→12
*T*_min_ = 0.977, *T*_max_ = 0.992	*l* = −17→17
2387 measured reflections	3 standard reflections every 200 reflections
1241 independent reflections	intensity decay: 1%

### Refinement

**Table:**

Refinement on *F*^2^	Secondary atom site location: difference Fourier map
Least-squares matrix: full	Hydrogen site location: inferred from neighbouring sites
*R*[*F*^2^ > 2σ(*F*^2^)] = 0.040	H atoms treated by a mixture of independent and constrained refinement
*wR*(*F*^2^) = 0.114	*w* = 1/[σ^2^(*F*_o_^2^) + (0.077*P*)^2^] where *P* = (*F*_o_^2^ + 2*F*_c_^2^)/3
*S* = 1.01	(Δ/σ)_max_ < 0.001
1241 reflections	Δρ_max_ = 0.15 e Å^−^^3^
154 parameters	Δρ_min_ = −0.12 e Å^−^^3^
0 restraints	Extinction correction: *SHELXL97* (Sheldrick, 2008), Fc^*^=kFc[1+0.001xFc^2^λ^3^/sin(2θ)]^-1/4^
Primary atom site location: structure-invariant direct methods	Extinction coefficient: 0.029 (5)

### Special details

**Table:**

Geometry. Bond distances, angles etc. have been calculated using the rounded fractional coordinates. All su's are estimated from the variances of the (full) variance-covariance matrix. The cell esds are taken into account in the estimation of distances, angles and torsion angles
Refinement. Refinement of *F*^2^ against ALL reflections. The weighted *R*-factor *wR* and goodness of fit *S* are based on *F*^2^, conventional *R*-factors *R* are based on *F*, with *F* set to zero for negative *F*^2^. The threshold expression of *F*^2^ > σ(*F*^2^) is used only for calculating *R*-factors(gt) *etc*. and is not relevant to the choice of reflections for refinement. *R*-factors based on *F*^2^ are statistically about twice as large as those based on *F*, and *R*- factors based on ALL data will be even larger.

### Fractional atomic coordinates and isotropic or equivalent isotropic displacement parameters (Å^2^)

**Table:**

	*x*	*y*	*z*	*U*_iso_*/*U*_eq_	
O1	−0.0723 (5)	0.48772 (18)	0.66689 (13)	0.1072 (11)	
N1	0.0516 (5)	0.2583 (3)	0.72595 (18)	0.0903 (13)	
C1	−0.0893 (6)	0.8175 (3)	0.2825 (2)	0.0979 (14)	
C2	−0.0848 (4)	0.7203 (2)	0.36079 (19)	0.0649 (10)	
C3	−0.1864 (4)	0.7310 (2)	0.4405 (2)	0.0668 (10)	
C4	−0.1798 (4)	0.6431 (2)	0.51329 (19)	0.0611 (8)	
C5	−0.0737 (3)	0.5373 (2)	0.50607 (16)	0.0507 (8)	
C6	0.0293 (3)	0.5256 (2)	0.42633 (17)	0.0562 (8)	
C7	0.0251 (4)	0.6161 (2)	0.35603 (17)	0.0612 (9)	
C8	−0.0684 (4)	0.4450 (2)	0.58631 (17)	0.0626 (9)	
C9	−0.0553 (3)	0.3074 (2)	0.56775 (17)	0.0515 (8)	
C10	0.0012 (4)	0.2205 (3)	0.63780 (18)	0.0597 (9)	
C11	0.0141 (4)	0.0914 (3)	0.6133 (2)	0.0665 (10)	
C12	−0.0327 (4)	0.0487 (2)	0.5261 (2)	0.0670 (10)	
C13	−0.0940 (4)	0.1319 (2)	0.45806 (19)	0.0632 (9)	
C14	−0.1042 (3)	0.2588 (3)	0.47965 (16)	0.0548 (8)	
H0B	0.065 (5)	0.203 (3)	0.766 (2)	0.094 (12)*	
H1A	−0.17140	0.88270	0.29780	0.1470*	
H1B	−0.12190	0.77690	0.22430	0.1470*	
H1C	0.02260	0.85510	0.27550	0.1470*	
H0A	0.029 (5)	0.336 (3)	0.743 (2)	0.082 (11)*	
H3A	−0.26180	0.79950	0.44540	0.0800*	
H4A	−0.24660	0.65500	0.56720	0.0730*	
H6A	0.10210	0.45570	0.42040	0.0670*	
H7A	0.09750	0.60730	0.30400	0.0730*	
H11A	0.05570	0.03360	0.65770	0.0800*	
H12A	−0.02340	−0.03760	0.51200	0.0800*	
H13A	−0.12750	0.10230	0.39880	0.0760*	
H14A	−0.14530	0.31490	0.43390	0.0660*	

### Atomic displacement parameters (Å^2^)

**Table:**

	*U*^11^	*U*^22^	*U*^33^	*U*^12^	*U*^13^	*U*^23^
O1	0.203 (3)	0.0665 (13)	0.0521 (10)	0.0088 (18)	−0.0032 (16)	−0.0151 (10)
N1	0.135 (3)	0.079 (2)	0.0570 (15)	−0.006 (2)	−0.0174 (16)	0.0122 (15)
C1	0.139 (3)	0.0648 (19)	0.090 (2)	−0.001 (2)	−0.013 (2)	0.0186 (16)
C2	0.0806 (19)	0.0466 (14)	0.0675 (16)	−0.0038 (15)	−0.0133 (16)	−0.0008 (13)
C3	0.0709 (17)	0.0412 (13)	0.0882 (19)	0.0067 (13)	−0.0089 (17)	−0.0106 (14)
C4	0.0668 (16)	0.0459 (13)	0.0706 (15)	−0.0026 (13)	0.0056 (14)	−0.0141 (13)
C5	0.0588 (15)	0.0404 (12)	0.0529 (13)	−0.0011 (11)	−0.0032 (12)	−0.0103 (10)
C6	0.0600 (15)	0.0458 (13)	0.0627 (14)	0.0027 (13)	−0.0049 (13)	−0.0089 (12)
C7	0.0746 (18)	0.0525 (14)	0.0566 (13)	−0.0028 (15)	0.0018 (14)	−0.0057 (12)
C8	0.082 (2)	0.0539 (15)	0.0518 (13)	−0.0015 (15)	−0.0025 (15)	−0.0097 (12)
C9	0.0549 (15)	0.0481 (13)	0.0515 (13)	−0.0006 (12)	0.0029 (12)	−0.0006 (11)
C10	0.0597 (16)	0.0637 (15)	0.0556 (14)	−0.0069 (14)	0.0048 (13)	0.0089 (13)
C11	0.0647 (17)	0.0523 (15)	0.0824 (18)	−0.0020 (14)	0.0066 (16)	0.0188 (14)
C12	0.0675 (18)	0.0423 (13)	0.0912 (19)	−0.0034 (13)	0.0102 (16)	−0.0006 (14)
C13	0.0690 (17)	0.0505 (14)	0.0700 (16)	−0.0059 (14)	−0.0022 (15)	−0.0095 (13)
C14	0.0588 (15)	0.0487 (12)	0.0570 (15)	−0.0003 (12)	−0.0055 (12)	−0.0020 (12)

### Geometric parameters (Å, °)

**Table:**

O1—C8	1.223 (3)	C10—C11	1.401 (4)
N1—C10	1.363 (4)	C11—C12	1.359 (4)
N1—H0B	0.82 (3)	C12—C13	1.382 (4)
N1—H0A	0.87 (3)	C13—C14	1.368 (4)
C1—C2	1.504 (4)	C1—H1A	0.9600
C2—C3	1.379 (4)	C1—H1B	0.9600
C2—C7	1.389 (4)	C1—H1C	0.9600
C3—C4	1.381 (4)	C3—H3A	0.9300
C4—C5	1.386 (3)	C4—H4A	0.9300
C5—C6	1.387 (3)	C6—H6A	0.9300
C5—C8	1.491 (3)	C7—H7A	0.9300
C6—C7	1.374 (3)	C11—H11A	0.9300
C8—C9	1.471 (3)	C12—H12A	0.9300
C9—C14	1.397 (3)	C13—H13A	0.9300
C9—C10	1.415 (4)	C14—H14A	0.9300
			
H0B—N1—H0A	120 (3)	C12—C13—C14	118.7 (2)
C10—N1—H0B	118 (2)	C9—C14—C13	122.5 (2)
C10—N1—H0A	118 (2)	C2—C1—H1A	109.00
C1—C2—C3	122.1 (2)	C2—C1—H1B	110.00
C1—C2—C7	120.8 (3)	C2—C1—H1C	109.00
C3—C2—C7	117.1 (2)	H1A—C1—H1B	109.00
C2—C3—C4	122.1 (2)	H1A—C1—H1C	109.00
C3—C4—C5	120.1 (3)	H1B—C1—H1C	109.00
C4—C5—C6	118.3 (2)	C2—C3—H3A	119.00
C4—C5—C8	118.7 (2)	C4—C3—H3A	119.00
C6—C5—C8	122.9 (2)	C3—C4—H4A	120.00
C5—C6—C7	120.8 (2)	C5—C4—H4A	120.00
C2—C7—C6	121.6 (2)	C5—C6—H6A	120.00
C5—C8—C9	120.3 (2)	C7—C6—H6A	120.00
O1—C8—C9	121.8 (2)	C2—C7—H7A	119.00
O1—C8—C5	117.9 (2)	C6—C7—H7A	119.00
C8—C9—C10	122.0 (2)	C10—C11—H11A	119.00
C8—C9—C14	119.9 (2)	C12—C11—H11A	119.00
C10—C9—C14	118.1 (2)	C11—C12—H12A	120.00
C9—C10—C11	118.2 (2)	C13—C12—H12A	120.00
N1—C10—C9	122.7 (3)	C12—C13—H13A	121.00
N1—C10—C11	119.1 (3)	C14—C13—H13A	121.00
C10—C11—C12	121.5 (3)	C9—C14—H14A	119.00
C11—C12—C13	120.9 (2)	C13—C14—H14A	119.00
			
C1—C2—C3—C4	−178.7 (3)	O1—C8—C9—C14	160.0 (3)
C7—C2—C3—C4	0.4 (4)	C5—C8—C9—C10	160.6 (3)
C1—C2—C7—C6	−179.2 (3)	C5—C8—C9—C14	−21.2 (4)
C3—C2—C7—C6	1.7 (4)	C8—C9—C10—N1	−1.8 (4)
C2—C3—C4—C5	−2.6 (4)	C8—C9—C10—C11	−178.4 (3)
C3—C4—C5—C6	2.6 (4)	C14—C9—C10—N1	−180.0 (3)
C3—C4—C5—C8	179.4 (2)	C14—C9—C10—C11	3.5 (4)
C4—C5—C6—C7	−0.5 (4)	C8—C9—C14—C13	179.6 (3)
C8—C5—C6—C7	−177.2 (2)	C10—C9—C14—C13	−2.2 (4)
C4—C5—C8—O1	−39.1 (4)	N1—C10—C11—C12	−179.3 (3)
C4—C5—C8—C9	142.1 (3)	C9—C10—C11—C12	−2.7 (5)
C6—C5—C8—O1	137.6 (3)	C10—C11—C12—C13	0.4 (5)
C6—C5—C8—C9	−41.2 (4)	C11—C12—C13—C14	1.0 (5)
C5—C6—C7—C2	−1.7 (4)	C12—C13—C14—C9	−0.1 (4)
O1—C8—C9—C10	−18.1 (5)		

### Hydrogen-bond geometry (Å, °)

**Table:**

*D*—H···*A*	*D*—H	H···*A*	*D*···*A*	*D*—H···*A*
N1—H0A···O1	0.87 (3)	2.08 (3)	2.723 (4)	131 (3)
N1—H0B···O1^i^	0.82 (3)	2.45 (3)	3.220 (4)	158 (3)
C11—H11A···O1^i^	0.93	2.53	3.319 (4)	143

Symmetry codes: (i) −*x*, *y*−1/2, −*z*+3/2.
